# Parity predicts biological age acceleration in post-menopausal, but not pre-menopausal, women

**DOI:** 10.1038/s41598-020-77082-2

**Published:** 2020-11-25

**Authors:** Talia N. Shirazi, Waylon J. Hastings, Asher Y. Rosinger, Calen P. Ryan

**Affiliations:** 1 Department of Anthropology, Pennsylvania State University, 421 Carpenter Building, University Park, PA 16802 USA; 2 Department of Biobehavioral Health, Pennsylvania State University, University Park, PA USA; 3 Department of Anthropology, Northwestern University, Evanston, IL USA

**Keywords:** Senescence, Evolutionary theory, Reproductive biology

## Abstract

Understanding factors contributing to variation in ‘biological age’ is essential to understanding variation in susceptibility to disease and functional decline. One factor that could accelerate biological aging in women is reproduction. Pregnancy is characterized by extensive, energetically-costly changes across numerous physiological systems. These ‘costs of reproduction’ may accumulate with each pregnancy, accelerating biological aging. Despite evidence for costs of reproduction using molecular and demographic measures, it is unknown whether parity is linked to commonly-used clinical measures of biological aging. We use data collected between 1999 and 2010 from the National Health and Nutrition Examination Survey (*n* = 4418) to test whether parity (number of live births) predicted four previously-validated composite measures of biological age and system integrity: Levine Method, homeostatic dysregulation, Klemera–Doubal method biological age, and allostatic load. Parity exhibited a U-shaped relationship with accelerated biological aging when controlling for chronological age, lifestyle, health-related, and demographic factors in post-menopausal, but not pre-menopausal, women, with biological age acceleration being lowest among post-menopausal women reporting between three and four live births. Our findings suggest a link between reproductive function and physiological dysregulation, and allude to possible compensatory mechanisms that buffer the effects of reproductive function on physiological dysregulation during a woman’s reproductive lifespan. Future work should continue to investigate links between parity, menopausal status, and biological age using targeted physiological measures and longitudinal studies.

## Introduction

Chronological age is a leading predictor of mortality, morbidity, and functional decline^1,2^. Despite the striking association between chronological age, lifespan, and health, individuals vary considerably in their rate of functional decline^3^. This variation—attributed to differences in the biological rate of deterioration or repair—is referred to as ‘biological age’, and is thought to reflect the cumulative effect of environmental exposures in combination with underlying genetic variation. Various proximate mechanisms have been proposed to modulate biological age acceleration, including insulin signaling^4^, oxidative stress^5^, inflammation^6^, epigenetic changes^7^, and telomere shortening^8^. Understanding the environmental, behavioral, and physiological factors that influence biological aging may inform policies and interventions that could help to mitigate their effects, thereby extending healthspan. Such policies and interventions will become increasingly important as the proportion of the global population over age 60 is expected to increase dramatically over the next 30 years^9^.

Environmental factors found to accelerate biological aging and functional decline include smoking^10^, obesity^10^, socioeconomic status^11^, and psychosocial stress^12^. Another lifestyle factor that may accelerate biological aging in women specifically is reproduction^13,14^. Reproduction in women is an energetically costly process, and is characterized by extensive changes in both form and function across numerous anatomical and physiological systems^15^. Pregnancy and breastfeeding are accompanied by shifts in immune function^16–18^, energy metabolism and storage^19,20^, blood pressure and volume^21,22^, and hormone levels and receptor expression^23^. Evolutionary theory predicts that these changes create functional or energetic constraints to somatic maintenance and repair, leading to accelerated biological age—a tradeoff referred to as ‘costs of reproduction’^24,25^.

Consistent with costs of reproduction in women, ever-parity has been linked to mortality from diabetes, cancer of the uterine cervix, gallbladder disease, kidney disease, hypertension, and all-cause mortality^26–29^. Similarly, women who give birth to more children are at higher risk of developing obesity, diabetes, hypertension and cardiovascular disease (CVD)^30,31^, as well as age-corrected all-cause mortality^28,32,33^, mortality related to cardiovascular disease^34^ and mortality related to kidney disease^35^. Notably, studies with the largest sample sizes (and presumably, the greatest statistical power) often reveal that parity exhibits a U-shaped association with all-cause mortality^32,33^ and CVD^34^, with lowest all-cause mortality and CVD rates observed at intermediate parity. The number of children or pregnancies has also been linked to multiple measures of cellular aging, including DNA damage and oxidative stress^36^, telomere length^13,14^, and DNA methylation age^14,37^. While most of these studies examine associations within Western populations, some evidence supporting costs of reproduction is seen in non-Western populations as well^14,36^.

Cellular measures of biological age such as telomere length and DNA methylation age may provide insights into the molecular processes linking reproduction to mortality and other health outcomes^7,38^, and may eventually serve as early indicators of the costs of reproduction in health and aging. However, ‘aging’ may refer to a wide range of processes that may occur at different times or at different speeds. For example, cellular measures of biological age that examine mitotic (e.g., telomere length) and non-mitotic (e.g., DNA methylation age) processes are often weakly correlated^14,39^. Similarly, both telomere length and DNA methylation age are poorly associated with measures of biological age implemented at the clinical level^40–43^. Thus, it has been suggested that different measures of cellular aging and cumulative system dysregulation index fundamentally different components of the aging process.

Clinical measures of biological age quantify changes in physiological integrity by combining information from multiple clinical biomarkers that collectively assess the function of major organ systems throughout the body. Such measures may be particularly relevant in light of the many physiological, immunological, and endocrinological changes that accompany reproduction in women^44^. Four composites of system integrity have been used to operationalize biological age and cumulative system dysregulation within the context of large-scale epidemiological studies in the United States: Homeostatic Dysregulation (HD)^45^, Levine Method Biological Age (LM)^46,47^, the Klemera–Doubal Method Biological Age (KDM)^46,48^, and allostatic load (AL)^49^. Previous work using a nationally representative sample of adults in the US from the National Health and Nutrition Examination Survey has found that HD, LM, KDM, and AL exhibit robust associations with physical functioning, cognition, hearing and vision, and with self-reports of health and functional disability^11,50^. Other population-based studies have found similar links between AL and both objective and subjective markers of physical functioning and general health^51^. Clinically-based measures may therefore provide an affordable and accessible alternative to cell-based measures for measuring systemic deterioration tied to costs of reproduction in women.

Here, we present nationally-representative estimates of the effect of parity (operationalized as number of live births) on four composites of system integrity indexing biological age and cumulative dysregulation. Using cross-sectional epidemiological data collected in the United States between 1999 and 2010, we test whether parity is associated with HD, KDM, LM, and AL while controlling for a range of covariates (e.g., smoking, obesity) known to modulate biological age. Although each measure utilizes the same panel of biomarkers, differences in scale construction provide a varied, multifactorial approach to the study of costs of reproduction on biological aging. Based on findings from the most highly powered prior studies of all-cause mortality and parity, we hypothesized a U-shaped relationship between parity and biological aging. Specifically, we predicted that accelerated biological aging would be most apparent in women with the lowest and the highest parity. We also leverage this powerful dataset for preliminary tests of whether relationships between parity and biological age are durable, such that they persist regardless of time since last birth, or transient, such that the effect of parity on biological age decreases as a function of time since last birth. Our findings have significant theoretical implications for our understanding of the relationship between parity and health, and of putative tradeoffs between reproductive and somatic effort in women.

## Materials and methods

### Data source

**Figure 1:**
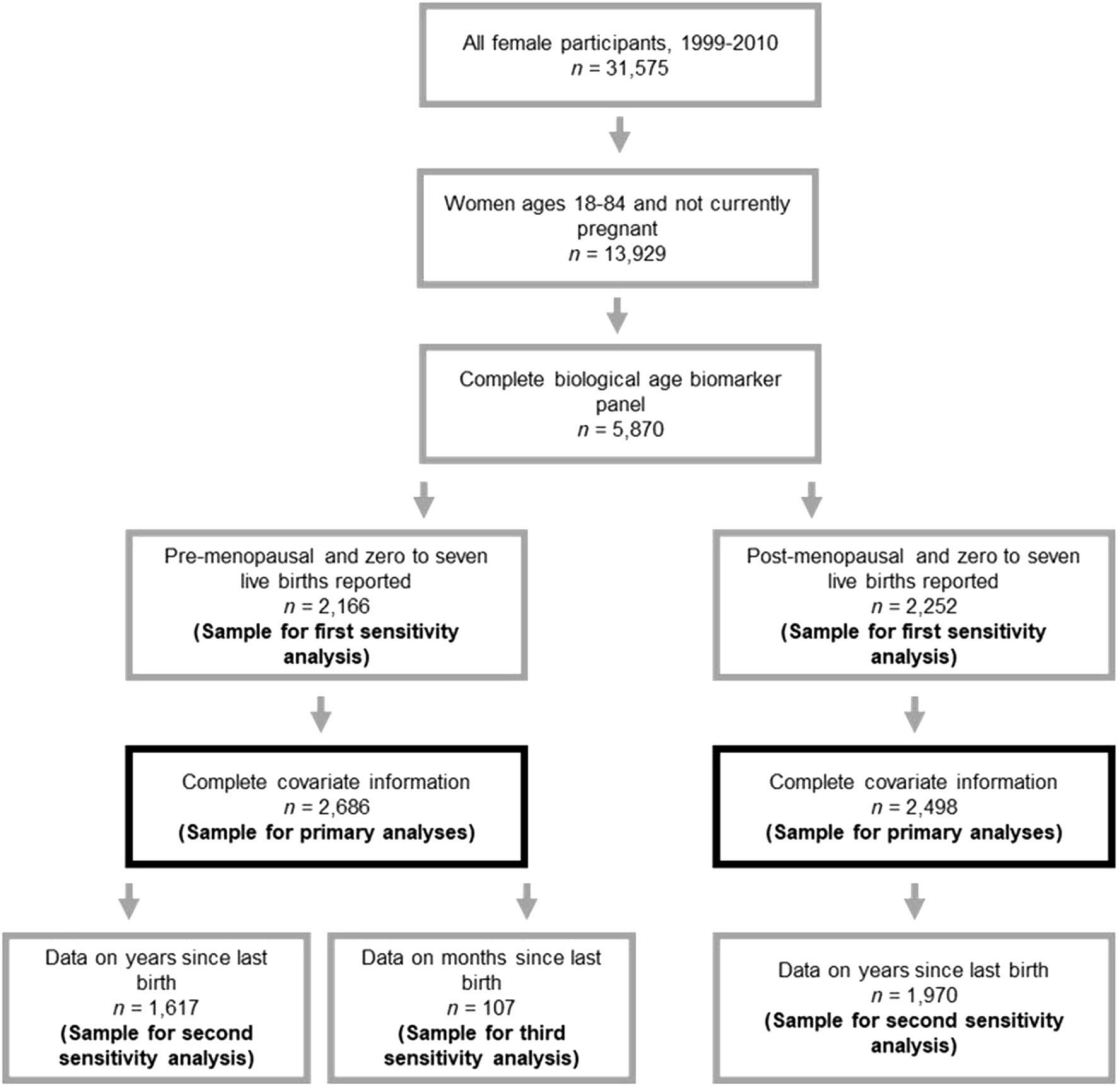
Flow chart illustrating sample stratification.

Data were collected as part of the Centers for Disease Control and Prevention’s National Health and Nutrition Examination Survey (NHANES). NHANES uses multistep cluster sampling, and assigns participants sample weights based on demographic variables such as self-identified race/ethnicity, age, and education; utilization of these sample weights in analyses enables estimation of population-level effects. Continuous sampling for NHANES began in 1999, and data is made publicly available in 2-year waves. Details of recruitment procedures and study design are available from the Centers for Disease Control and Prevention^52^. Women sampled between 1999 and 2010 are included in the present analyses, as not all the data necessary to construct the biological aging measures (i.e. C-reactive protein) were released for cycles following the 2009–2010 cycle at the time of writing this manuscript. Furthermore, women missing information on any covariate included in analyses were excluded from the sample. A flowchart detailing sample stratification can be found in Fig. 1, and sample demographic information is presented in Table 1.

**Table 1:** Sample demographic characteristics (*n* = 4418), National Health and Nutrition Examination Survey, 1999–2010. Means, standard errors (SE), and percentages represent nationally-representative estimates based on adjustment for complex survey design, survey nonresponse, non-coverage, and complex survey design. Unless otherwise noted, p-values reflect tests of difference via *t* test or Chi-Square as appropriate.

	Pre-menopausal (*n* = 2166)	Post-menopausal (*n* = 2252)	p-value
Mean age (SE, range)	34.92 (0.26, 20–61)	63.50 (0.30, 41–84)	< 0.001
Mean BMI (SE, range)	28.18 (0.18, 15.6–71.3)	29.04 (0.16, 14.7–57.6)	< 0.001
Mean FIPR (SE, range)	2.86 (0.05, 0–5)	3.03 (0.05, 0–5)	< 0.001
Smoking (n, %)			< 0.001
Never	1367 (59.2%)	1278 (52.6%)	
Past	290 (16.4%)	659 (31.1%)	
Current	509 (24.4%)	315 (16.3%)	
Education (n, %)			< 0.001
Less than high school	489 (22.6%)	682 (20.8%)	
High school or equivalent	465 (21.9%)	617 (29.9%)	
Some college or AA degree	744 (36.4%)	593 (28.7%)	
College graduate or above	468 (19.1%)	360 (20.6%)	
Race/ethnicity (n, %)			< 0.001
Non-Hispanic white	1007 (46.5%)	1309 (58.1%)	
Non-Hispanic black	445 (20.5%)	396 (17.6%)	
Hispanic	626 (28.9%)	488 (21.7%)	
Other	88 (4.1%)	59 (3.3%)	
Mean number of live births (SE, range)	1.60 (0.04, 0–7)	2.58 (0.04, 0–7)	< 0.001
Ever-parity (n, %)			< 0.001
Nulliparous	534 (27.6%)	237 (11.1%)	
Parous	1632 (72.4%)	2015 (88.9%)	
LM biological age	30.42 (0.28, 4.7–81.3)	59.23 (0.37, 26.0–103.6)	0.002^†^
LM biological age acceleration	− 0.57 (0.13, − 14.1–32.3)	− 0.69 (0.18, − 14.7–47.9)	
Homeostatic dysregulation	3.09 (0.01, 1.5–4.8)	3.25 (0.01, 1.5–5.3)	0.696^†^
Homeostatic dysregulation	− 0.04 (0.01, − 1.6–1.62)	− 0.03 (0.01, − 1.9–2.1)	
KDM biological age	31.77 (0.31, 0.6–111.6)	58.16 (0.41, 17.1–147.3)	< 0.001^†^
KDM biological age acceleration	− 1.14 (0.24, − 25.7–83.85)	− 0.15 (0.36, − 34.7, 83.2)	
Allostatic load	0.21 (0.003, 0.0–0.8)	0.30 (0.01, 0.0–0.9)	0.307^†^
Allostatic load acceleration	− 0.02 (0.004, − 0.3–0.54)	− 0.01 (0.01, − 0.4–0.6)	

^†^p− values from linear regression models adjusted for the following variables: chronological age, body mass index, federal income-to-poverty ratio, smoking, education, and self-identified race/ethnicity.

To assess the representativeness of participants with complete biomarker information, we compared the subset of non-pregnant women aged 18–84 with complete biomarker data (*n* = 5870) to all non-pregnant women aged 18–84 in NHANES 1999–2010 (*n* = 13,929). The two samples were similar in age, ethnicity, educational attainment, income, smoking status, menopausal status, and number of live births. However, the sample with complete biomarker data was significantly more likely to have ever been pregnant. Comparative demographics and associated tests of difference are reported in ESM Table I.

### Reproductive health and parity data

**Figure 2:**
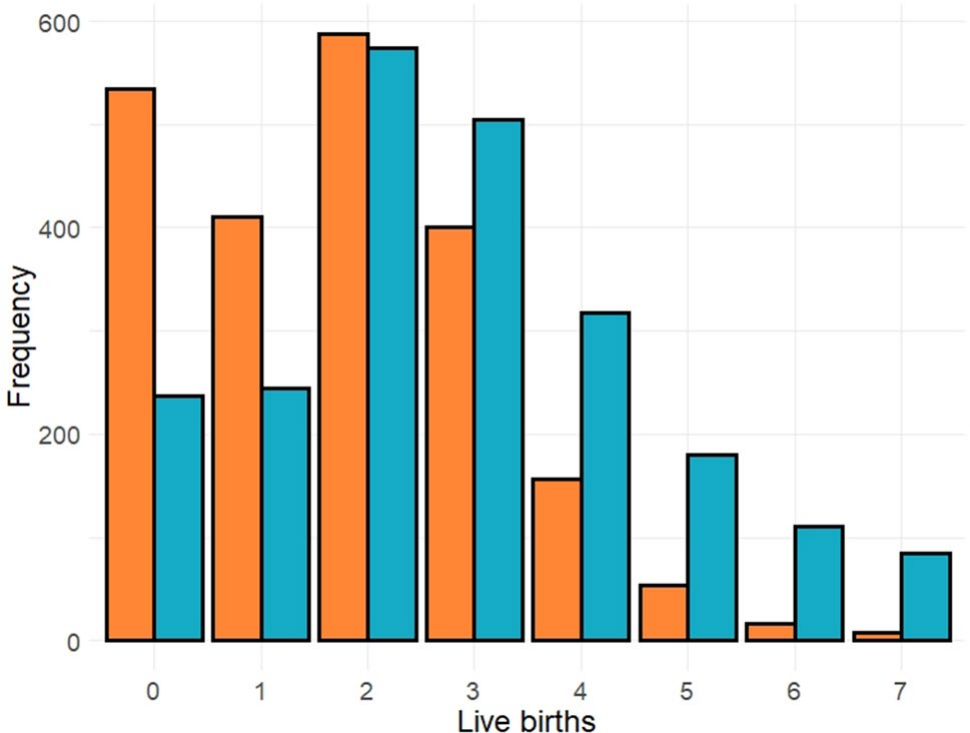
Distribution of live births for pre-menopausal (orange bars; *n* = 2166) and post-menopausal (blue bars; *n* = 2252).

Women completed a computer-assisted questionnaire on their reproductive health history. Women reported whether they were currently pregnant, if they have ever been pregnant, how many pregnancies resulted in a live birth (if applicable; NHANES items RHD170 and RHQ171), whether they had regular periods over the last 12 months, and their reason for not having regular periods over the last 12 months (if applicable). As previous work has suggested that current pregnancy modulates certain measures of biological age^14^, women who self-reported currently being pregnant were excluded from analyses (NHANES item RIDEXPRG; *n* = 1417 out of all women between 18 and 84). Due to the small number of women with complete covariate information who reported 8 or more live births (*n* = 137), these women were excluded from analyses. The frequency distribution of live births for women included in our analyses is displayed in Fig. 2. NHANES does not collect fine-grained data about pregnancies that do not result in live births, rendering it impossible to estimate the length of each pregnancy, and concomitantly, the total physiological cost of each pregnancy. Further, approximately 30% of implantations end in natural miscarriage^53^, making number of recognized pregnancies a more imprecise measure of physiological investment in reproduction as compared to number of live births. As a result, we chose to use number of live births rather than number of pregnancies. Women who reported a prior live birth indicated their age at last live birth across all survey cycles. Because responses to this question were bottom-coded at 14 and top-coded at 45 for some cycles, we limited our analysis to women who reported an age of last live birth between 15 and 44. Starting in the 2007–2008 cycle, NHANES added a question on the number of months since last live birth for women who reported up to a 2 year difference between their current age and age of last birth.

Women were categorized as being pre-menopausal if they reported having regular periods over the last 12 months, if they reported not having regular periods because of a reason other than menopause, or if they were younger than 41. A lower limit of 41 was chosen because the average age of menopause in the US is 51, and perimenopause may last up to 10 years for some women^54^. Women were categorized as being post-menopausal if they were older than 61, or if they reported not having regular periods over the last 12 months because of menopause.

### Biological aging measures

All composite measures of biological aging were constructed using the following 9 biomarkers: albumin, creatinine, glucose, log-transformed C-reactive protein (CRP), lymphocyte percent, mean cell volume, red blood cell distribution width, alkaline phosphatase, and white blood cell count. Where appropriate, female participants from NHANES III, for which data collection ran between 1988 and 1994, were used as the reference sample for the construction of the biological aging measures employed here. Serum creatinine values from NHANES III and NHANES 1999–2004 continuous panels were adjusted according to published recommendations^55^.

*Homeostatic Dysregulation (HD)* is a measure of Mahalanobis distance^56^, quantifying the deviation of a participant’s physiology from a young, healthy reference norm. Following previous work^11^, we defined our reference population as non-pregnant women from NHANES III aged 20–30 who were not obese (BMI < 30) and for whom all biomarkers fell within the clinically normal range for their age and sex (*n* = 481, see ESM Tables II–IV). Biomarker values from the reference population were standardized and used to compute a biomarker variance–covariance matrix (ESM Table IV). Biomarker raw means, raw standard deviations, and the standardized-biomarker variance–covariance matrix are implemented within the Mahalanobis distance equation^56^ to form the homeostatic dysregulation (HD) algorithm: $HD = \sqrt {\left( {\vec{v} - \vec{\mu }} \right)^{T} * S^{{ - 1}} *\left( {\vec{v} - \vec{\mu }} \right) }$. Here, *v* is a vector of biomarker values for a participant in the analysis sample; *u* is a vector of biomarker means in the training sample, and *S* is the standardized-biomarker variance–covariance matrix. As HD in the full sample was significantly skewed, natural log-transformed HD was used as the outcome variable in all analyses.

*Klemera–Doubal Method (KDM) Biological Age* is computed using the Klemera–Doubal equation^48^, which extracts information from individual regressions of chronological age onto *m* biomarkers: $KDM=\frac{\sum_{j=1}^{m}\left({x}_{j}-{q}_{j}\right)\frac{{k}_{j}}{{s}_{j}^{2}}+\frac{CA}{{s}_{BA}^{2}}}{{\sum }_{j=1}^{m}{\left(\frac{{k}_{j}}{{s}_{j}}\right)}^{2}+\frac{1}{{s}_{BA}^{2}}}$. Here, *x*_*j*_ is the value of biomarker *j* measured for an individual in the analytical sample and *CA* is their chronological age. For each biomarker *j*, the parameters *q* (intercept), *k* (slope), and *s* (root mean squared error) are estimated from a regression of chronological age onto the biomarker in the reference population. *sBA* is a scaling factor equal to the square root of the variance in chronological age explained by the biomarker panel in the reference population^46^ (Eq. 5). Following previous work^46^, we formed our reference population from non-pregnant women in NHANES III aged 30–75 (*n* = 5453, see ESM Tables V and VI). An individual's KDM Biological Age corresponds to the average chronological age at which their physiology would be observed in the reference population.

*Levine Method (LM) Biological Age* is computed from a multivariate analysis of mortality hazards using NHANES III data^46,47^. Herein, a multivariate Gompertz model of mortality hazard is fit to the selected biomarkers and chronological age to form a predicted hazard of mortality called a “mortality score”. This mortality score is converted to a biological age value using a second univariate Gompertz regression of the mortality hazard onto chronological age. In this manner, the LM biological age is interpretable as the chronological age at which an individual’s physiology-based risk for mortality would be approximately normal in the reference population. We applied published parameters from Liu and colleagues’ original work^47^ to compute LM biological age for participants in our sample.

*Allostatic Load (AL)* is computed as the proportion of biomarker values for which a participant is at risk. In accordance with recommendations from a review of AL implementation in NHANES^57^, we defined risk as residing within the highest quartile of a given biomarker’s distribution within the sample of nonpregnant women aged 18–84 with complete biological age biomarker data, excepting albumin for which risk was defined as residing in the lowest quartile (*n* = 5870; ESM Table VII). In this manner, the number of biomarkers for which a participant is at risk is divided by the total number of biomarkers in the panel to calculate a final allostatic load score with values ranging from 0 to 1.

All four biological aging measures were computed using the same panel of 9 biomarkers. These biomarkers were selected based upon their inclusion in the LM biological age algorithm, which utilized machine-learning analysis to select the most parsimonious panel of biomarkers for mortality prediction. The use of common biomarkers ensures the different measures are indexing the same physiological processes. Differences in the analytical approach and statistical operations leading to the final composite measure reflects different approaches toward the conceptualization of biological age. For HD, biological age is conceptualized as deviation from an ideal physiological state attained in one’s 20s. For KDM, biological age is conceptualized as the average change in physiology that occurs with increasing chronological age. Building upon this, LM captures the increased risk in mortality that accompanies physiological changes occurring with age. Finally, AL conceptualizes aging as the accumulation of changes that become impactful only once they reach a critical threshold. Biomarker and biological age summary statistics for the final analytical sample (*n* = 4418) are provided in ESM Table VIII.

**Figure 3:**
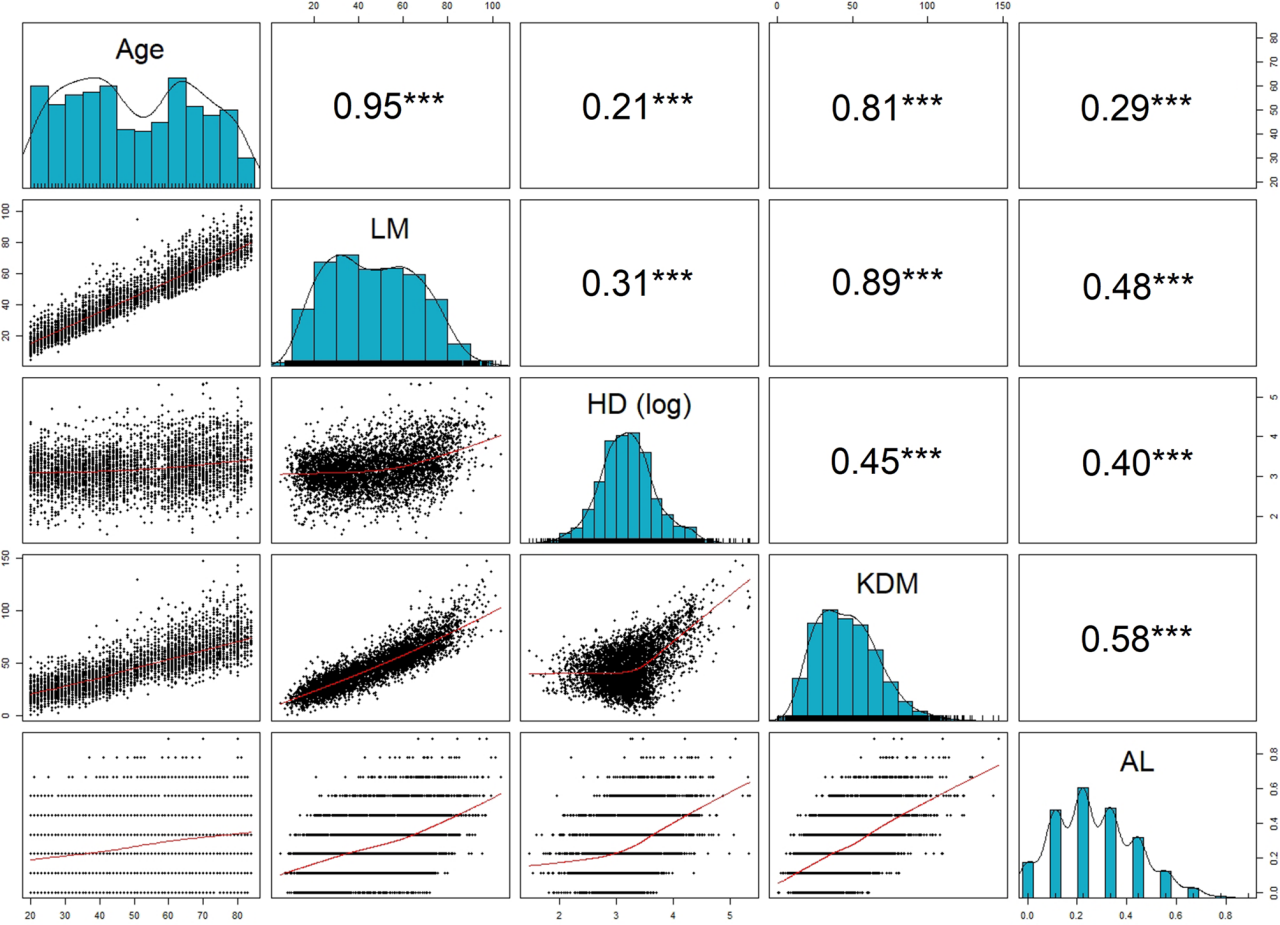
Associations between measures of chronological and biological age employed in the present study, National Health and Nutrition Examination Survey 1999–2010 (*n* = 4418). Numbers represent Pearson correlation coefficients. Note: *** p < 0.001; LM, Levine Method; HD, homeostatic dysregulation; KDM, Klemera-Doubal Method; AL, allostatic load.

Univariate distributions, bivariate distributions, and Pearson correlations coefficients for age, LM, log-transformed HD, and KDM are displayed in Fig. 3. As expected, all four measures of biological age were significantly correlated with chronological age, and all four measures of biological age were significantly correlated with each other.

### Covariates

Self-reported race/ethnicity^58^, socioeconomic status (SES)^59,60^, and smoking^10^ moderate the relationship between chronological age and biological aging. Self-reported race/ethnicity was categorized as non-Hispanic (NH) white, NH black, Hispanic, and ‘other’ (NHANES item RIDRETH1). SES was indexed by educational attainment (NHANES item DMDEDUC2) and federal income-to-poverty ratio (FIPR; NHANES item INDFMPIR as calculated per Department of Health and Human Services guidelines). Height and weight were measured by an NHANES examiner, and BMI was calculated as weight (kg) divided by height (meters squared; NHANES item BMXBMI). As prior work has shown that BMI exhibits a U-shaped curve with negative health outcomes^61^, our models included both linear and quadratic terms for BMI. On the basis of responses to a computer-assisted questionnaire on smoking habits, women were classified as never, past, or current smokers. To better isolate the effect of parity and biological age, our primary models controlled for the aforementioned covariates.

### Statistical analyses

All analyses were performed in R using the *survey* package, which supports functionality for analyzing data from complex survey designs. To facilitate accessibility of our methods, we also performed all analyses in Stata version 16.1. R scripts, Stata scripts, and data files have been uploaded online and can be found at https://osf.io/b2jft/.

We followed all NHCS guidelines for the analysis of NHANES data^62^. As the survey weights relevant to the smallest sample subpopulation for which all data are available should be used, we used mobile examination center (MEC) weights to adjust for complex survey design, oversampling, non-coverage, day of the week, and survey nonresponse to compute nationally representative estimates^63,64^. Per NHANES analytical guidelines for combining data across cycles, 12-year MEC weights were calculated using the NHANES-provided variables WTMEC4YR and WTMEC2YR as follows: $WTMEC12YR = \frac{1}{3}*WTMEC4YR\;for\;the\;1999 - 2000\;and\;2001 - 2002\;cycles$ and $WTMEC12YR = \frac{1}{6}*WTMEC2YR\;for\;all\;subsequent\;\;cycles$.

We estimated multiple linear regression models to examine the association of number of live births on biological age when controlling for chronological age, self-reported race/ethnicity, educational attainment, FIPR, BMI, and smoking. To focus on biological aging, we conducted analyses using versions of each biological age measure after adjustment for chronological age, computed as the residuals of each measure regressed only chronological age. Following adjustment, biological aging measures were no longer correlated with chronological age (ESM Table IX). Separate models were estimated for LM, log-transformed HD, KDM, and AL. Because we estimated four regressions (one per outcome measure) for each set of analyses for each analytical subset, statistical significance was set to *p* < 0.0125 (0.05/4)^65^.

We estimated both linear and quadratic terms for number of live births, as it has been previously suggested that the number of live births may exert quadratic, rather than linear, effects on morbidity and mortality^32–34^. As higher values correspond to more advanced biological age across all biological aging measures, a positive linear effect suggests a higher number of live births is associated with a higher biological age. A positive quadratic effect would suggest a convex (or U-shaped) shape to the fitted curve, while a negative quadratic effect would suggest a concave shape to the fitted curve. As prior work suggests that costs of reproduction should be the most apparent after menopause^44,66^, models were estimated separately pre-menopausal and post-menopausal women. Equations for each regression are provided in ESM Text 1.

**Figure 4:**
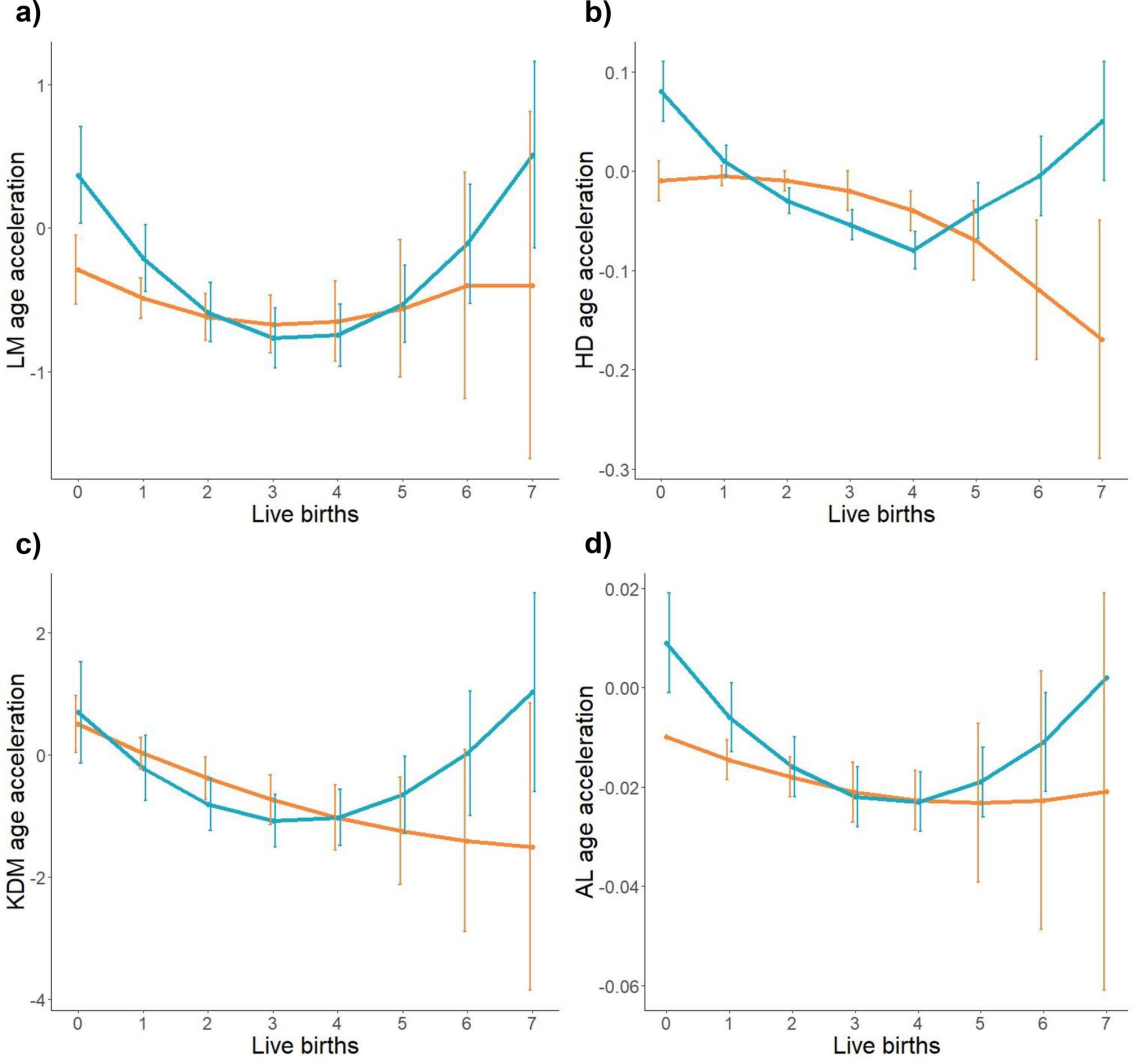
Predicted values and 95% confidence intervals derived from primary models for Levine Method (LM) age acceleration (**A**), Homeostatic Dysregulation (HD) acceleration (**B**), Klemera-Doubal Method (KDM) age acceleration (**C**), and Allostatic Load (AL) age acceleration (**D**) among pre-menopausal women (orange lines) and post-menopausal women (blue lines), National Health and Nutrition Examination Survey (*n* = 4418). Note: Figure generated using marginal standardization adjusted for the distribution of age, BMI, FIPR, smoking, education, and race/ethnicity.

Values for Fig. 4 were generated using Stata through post-estimation marginal standardization commands for regressions adjusting for the distribution of other covariates^67^. The y-axes in these figures represent the extent to which chronological age deviates from biological age. For each measure, this presents the difference between observed biological age and biological age predicted by chronological age (i.e., the residual of each biological aging measure regressed onto the chronological age). In all four cases, positive values indicate aging acceleration (biological age > chronological age) while negative values indicate age deceleration (biological age < chronological age).

### Sensitivity analyses

We conducted a series of follow-up regressions to probe the robustness of our primary analyses. First, we repeated the multiple linear regressions exactly as described above, including only chronological age as a covariate. This was done to ensure the relationship between variables included in our primary analyses and biological age were so strong as to mask putative relationships between parity and biological age. For example, in our sample BMI was significantly, positively correlated with LM and KDM (*r* = 0.29 and 0.28, respectively; *p* < 0.001).

We then estimated a second and third set of sensitivity analyses, with time since last birth used to create additional model terms. We did not include time since last birth in our primary analyses for two reasons. First, models including time since last birth by default eliminate all nulliparous women, rendering us unable to calculate estimates for the effect of nuliparity for nulliparous women. Second, data on time since last birth were missing for a significant portion of our sample. In these models, we assessed the extent to which effects of parity may be durable and accumulate over time, or transient and only present in the postnatal period. To assess potential durable effects of parity on biological aging, years since last birth was calculated for women across all survey cycles as age of last live birth subtracted from current chronological age. To assess potential transient effects data on months since last birth was available for women sampled in the 2007–2008 and 2009–2010 cycles. We estimated one set of regressions exactly as described above for our primary analyses, and added terms for the main effect of years since last birth and interactions between years since last birth and parity (sensitivity analysis 2). We then estimated additional set of regressions exactly as described above for our primary analyses and added terms for the main effect of months since last birth and interactions between months since last birth and parity (sensitivity analysis 3); however, this analysis was conducted in pre-menopausal women only since data on months since last birth were not available for any post-menopausal women.

### Ethical approval

All sampling procedures were approved through the National Center for Health Statistics (NCHS) Ethics Review Board and complied with all relevant human subjects protections and regulations, and all participants provide informed consent before sample collection and interviews.

## Results

### Differences between pre-menopausal and post-menopausal women

Demographic differences and differences in biological age acceleration are presented in Table 1. When adjusting for demographic differences, pre-menopausal women exhibited significantly lower LM and KDM biological age acceleration relative to post-menopausal women.

### Pre-menopausal women

The linear effect of number of live births and squared term, or quadratic effect, of live births was not significant in any primary model in pre-menopausal women (*n* = 2166; see Table 2; Fig. 4). Sample sizes for our sensitivity analyses controlling for chronological age only were slightly larger (*n* = 2686), as less participants were excluded due to missing covariate information. Similar to our primary analyses, the main effects of live births (both linear and quadratic terms) were not significant across all measures of biological age (Table 2). Repetition of these analyses in the primary analytical sample yielded the same pattern of results. Of the 2166 pre-menopausal women in our primary analyses, data on years since last live birth were available for 1617. The average years since last live birth was 8.87 (SE = 0.19). After correcting for multiple comparisons, the main effect of years since last live birth was not significant in any model, nor were any of the interaction terms between years since last live birth and parity (Table 2).

**Table 2:** Multiple linear regression examining the durable and transient effects of number of live births on biological age acceleration for pre-menopausal women only, National Health and Nutrition Examination Survey 1999–2010. Values represent coefficient estimates and 95% confidence intervals.

	LM	HD (log)	KDM	AL
**Primary model (n = 2166)**^†^				
Live births (linear)	− 0.24 (− 0.70, 0.22)	0.02 (− 0.03, 0.06)	− 0.51 (− 1.53, 0.51)	− 0.01 (− 0.02, 0.01)
Live births (quadratic)	0.04 (− 0.06, 0.13)	− 0.01 (− 0.02, 0.004)	0.03 (− 0.18, 0.24)	0.001 (− 0.002, 0.003)
**Sensitivity analysis 1 (n = 2686)**^††^				
Live births (linear)	0.03 (− 0.46, 0.52)	0.01 (− 0.03, 0.05)	− 0.24 (− 1.15, 0.67)	− 0.004 (− 0.02, 0.01)
Live births (quadratic)	0.06 (− 0.04, 0.17)	− 0.003 (− 0.01, 0.01)	0.05 (− 0.13, 0.24)	0.002 (− 0.001, 0.01)
**Sensitivity analysis 2 (n = 1617)**^†^				
Live births (linear)	− 0.04 (− 1.66, 1.57)	0.08 (− 0.06, 0.22)	− 1.34 (− 4.24, 1.56)	− 0.04 (− 0.08, 0.01)
Live births (quadratic)	0.01 (− 0.26, 0.29)	− 0.02 (− 0.04, 0.001)	0.10 (− 0.40, 0.60)	0.005 (− 0.002, 0.01)
Years since last birth	0.02 (− 0.14, 0.19)	− 0.01 (0.02, 0.01)	− 0.004 (− 0.36, 0.36)	− 0.002 (− 0.01, 0.003)
Live births (linear) × years since last live birth	0.03 (− 0.10, 0.17)	− 0.001 (− 0.01, 0.01)	0.02 (− 0.27, 0.32)	0.002 (− 0.003, 0.006)
Live births (quadratic) × years since last live birth	− 0.01 (− 0.03, 0.02)	0.001 (− 0.002, 0.003)	0.006 (− 0.05, 0.06)	− 0.0002 (− 0.0009, 0.0006)
**Sensitivity analysis 3 (n = 107)**^†^				
Live births (linear)	− 6.63 (− 13.19, − 0.07) *	0.25 (− 0.39, 0.90)	− 2.70 (− 14.83, 9.43)	− 0.06 (− 0.21, 0.08)
Live births (quadratic)	1.15 (0.14, 2.17) *	− 0.02 (− 0.12, 0.08)	0.66 (− 1.49, 2.82)	0.02 (− 0.01, 0.04)
Months since last live birth	− 1.07 (− 1.81, − 0.34) *	0.05 (− 0.04, 0.14)	− 0.71 (− 2.06, 0.65)	− 0.01 (− 0.02, 0.01)
Live births (linear) × months since last live birth	0.60 (0.14, 1.05) *	− 0.04 (− 0.10, 0.02)	0.23 (− 0.72, 1.17)	− 0.001 (− 0.01, 0.01)
Live births (quadratic) × months since last live birth	− 0.09 (− 0.15, − 0.02) *	0.01 (− 0.003, 0.014)	− 0.03 (− 0.18, 0.11)	0.0003 (− 0.001, 0.002)

^†^Models were adjusted for the following variables: chronological age, body mass index, federal income-to-poverty ratio, smoking, education, and self-identified race/ethnicity.

^††^Model was adjusted for chronological age only.

*p < 0.05, **p < 0.01, ***p < 0.001; values in bold represent effects significant after multiple comparison correction at α = (0.05/4) = 0.0125.

Our sample size for analyses including months since last live birth (*n* = 107) was significantly limited by the fact that this subsample excluded all post-menopausal women, and excluded women sampled prior to this question being added in the 2007–2008 cycle. On average, women with valid responses to this question gave birth 10.7 months ago (SE = 0.63). After correcting for multiple comparisons, the main effects of months since last live birth and parity was not significant in any model, nor were any of the interaction terms between months since last live birth and parity (Table 2). These results should be interpreted with caution given the small sample size.

### Post-menopausal women

Primary models in post-menopausal women revealed a significant linear effect of live births on biological aging indexed by LM, HD, and AL; the linear effect of live births on KDM was not significant after correction for multiple comparisons (*n* = 2252; Table 3). After correcting for multiple comparisons, the quadratic effect of parity on biological aging was significant for all measures but KDM. Sample sizes for our sensitivity analyses controlling for chronological age only were slightly larger (*n* = 2498). Similar trends were observed in the first set of sensitivity analyses, wherein the linear effect of live births was significantly associated with LM, HD, and AL. Moreover, the quadratic effect was significant for all four measures, giving rise to the anticipated U-shape for the overall relationship between parity and biological aging (shown in blue on Fig. 4). Repetition of these analyses in the primary analytical sample yielded the same pattern of results. Of the 2252 post-menopausal women in our primary analyses, data on years since last birth were available for 1970. The average years since last birth was 36.09 (SE = 0.25). After correcting for multiple comparisons, the main effect of years since last live birth was not significant in any model, nor were any of the interaction terms between years since last live birth and parity (Table 3).

**Table 3:** Multiple linear regression examining the durable and transient effects of number of live births on biological age acceleration for post-menopausal women only, National Health and Nutrition Examination Survey 1999–2010. Values represent coefficient estimates and 95% confidence intervals. *Notes:* *p < 0.05, **p < 0.01, ***p < 0.001; values in bold represent effects significant after multiple comparison correction at α = (0.05/4) = 0.0125.

	LM	HD (log)	KDM	AL
**Primary model (n = 2252)**^†^				
Live births (linear)	− **0.68 (**− **1.11, **− 0.25)**	− **0.07 (**− **0.11, **− 0.04)***	− 1.07 (− 2.12, − 0.02)*	− **0.02 (**− **0.03, **− 0.01)**
Live births (quadratic)	0.10 (0.03, 0.17)**	0.010 (0.004, 0.02)**	0.16 (− 0.01, 0.33)	0.002 (0.001, 0.004)*
**Sensitivity analysis 1 (n = 2498)**^††^				
Live births (linear)	− **0.80 (**− **1.30, **− 0.30)**	− **0.08 (**− **0.12, **− 0.04)***	− 1.10 (− 2.10, − 0.10)*	− **0.02 (**− **0.03, **− 0.01)**
Live births (quadratic)	0.17 (0.09, 0.25)***	0.013 (0.007, 0.02)***	0.23 (0.08, 0.39)**	0.004 (0.002, 0.01)***
**Sensitivity analysis 2 (n = 1.970)**^†^				
Live births (linear)	− 0.27 (− 2.76, 2.22)	− 0.21 (− 0.45, 0.04)	− 2.75 (− 8.56, 3.06)	− 0.02 (− 0.10, 0.05)
Live births (quadratic)	0.06 (− 0.33, 0.46)	0.03 (− 0.01, 0.06)	0.50 (− 0.33, 1.32)	− 0.005 (− 0.005, 0.015)
Years since last birth	− 0.01 (− 0.11, 0.08)	− 0.002 (− 0.01, 0.01)	− 0.02 (− 0.32, 0.27)	0.001 (− 0.003, 0.004)
Live births (linear) × years since last live birth	0.005 (− 0.07, 0.08)	0.004 (− 0.002, 0.01)	0.07 (− 0.10, 0.24)	0.0003 (− 0.002, 0.002)
Live births (quadratic) × years since last live birth	− 0.001 (− 0.01, 0.01)	− 0.001 (− 0.002, 0.000)	− 0.01 (− 0.04, 0.01)	− 0.0001 (− 0.0004, 0.0002)

^†^Models were adjusted for the following variables: chronological age, body mass index, federal income-to-poverty ratio, smoking, education, and self-identified race/ethnicity.

^††^Model was adjusted for chronological age only.

*p < 0.05, **p < 0.01, ***p < 0.001; values in bold represent effects significant after multiple comparison correction at α = (0.05/4) = 0.0125.

## Discussion

We tested putative physiological costs of reproduction using four validated measures of biological age and system integrity among a nationally-representative sample of US women of reproductive and post-reproductive age. Based on epidemiological studies, we hypothesized a U-shaped relationship between parity and biological age. Controlling for lifestyle, health-related, and demographic factors, we found evidence that parity is associated with all four measures of biological age among post-menopausal women, although this relationship was not significant for KDM after controlling for multiple comparisons. The relationship between parity and biological age in post-menopausal women is most consistent with a U-shaped pattern, with biological age acceleration reaching a minimum at 3–4 live births and more pronounced aging at either extreme. Parity was not associated with any measure of biological aging among pre-menopausal women. Our study represents the first application of biological age composites indexing system integrity (LM, HD, KDM, AL) to quantify costs of reproduction in both pre- and post-menopausal women, and may help elucidate some of the physiological processes bridging cellular and epidemiological findings relating parity with health and lifespan in women.

Our findings are broadly consistent with evolutionary theory^68^, studies of cellular aging and reproduction^14^, and epidemiological studies^32,69^. Despite evidence supporting costs of reproduction in women from each of these research domains, the physiological processes underlying such costs are still unclear. The composite measures used in our analysis were constructed using clinical markers of metabolic health (glucose), kidney and liver function (creatinine, albumin, alkaline phosphatase), anemia and/or red blood cell disorders (mean cell volume, red blood cell distribution width), and immune function and inflammation (CRP, lymphocyte percent, white blood cell count). Despite each composite measure weighting these clinical markers differently, we found evidence for a relationship between parity and accelerated biological aging in post-menopausal women using all four composite measures. This suggests that parity is associated with dysregulation across a broad range of physiological systems in post-menopausal women. Such broad physiological consequences of parity is consistent with the widespread metabolic, immunological, and endocrinological changes that accompany pregnancy and lactation, as well as the diverse disease risks that are both positively and negatively associated with parity in women^26^. Additional research focusing on the effect of parity on the individual clinical markers used in our composite measures will be an important next step in resolving the relative contributions of different physiological processes to parity-induced biological age acceleration in women.

We also found evidence for a non-linear increase in biological age with parity, as evidenced by the quadratic term in our models. This is consistent with several large meta-analyses examining the relationship between parity and cardiovascular disease^34^ and all-cause mortality^32^. The fact that these and other large studies^27,70^ show clear non-linear curves similar to those reported here gives us confidence in the robustness of our findings. Nevertheless, the reasons for the U-shaped relationship between parity and health and mortality are still unclear. In some cases, higher mortality among nulliparous women may be tied to selection effects whereby women with long-term illnesses or health problems may be less likely to marry or bear children^70^. Higher mortality among women bearing one child may similarly relate to long-term health issues, including those related to their first pregnancy^70^. Women with no children or only one child may also experience lower levels of social support^71^, which could have negative consequences on health later on in life^72^. Additional work to help disentangle the social and environmental factors that are associated with nulliparity or single parity is warranted.

Another explanation for the non-linear relationship between parity and biological age described here may include the interaction of countervailing physiological changes on our measures of biological aging. For example, risk increases with parity for many (i.e. CVD, diabetes, kidney cancer, hypertension, gallbladder cancer), but not all diseases (i.e. respiratory disease, breast, ovarian, endometrial cancer)^26,31^. The non-linear relationship we observe between parity and biological age may therefore reflect the cumulative effect of both beneficial and harmful physiological accommodations necessary for reproduction in women, no doubt also mediated by individual risk factors tied to genetic variation, environment, or lifestyle.

That parity was not associated with biological age in pre-menopausal women, along with the finding that time since last birth did not predict biological age acceleration in either pre- or post-menopausal women supports the hypothesis that the effects of parity are durable, and not simply short-term physiological changes associated with pregnancy and breastfeeding. The reasons for our findings being limited to post-menopausal women are unclear, but are in accordance with research in both historical populations^66,73^ and contemporary epidemiological studies, where the relationship between parity and disease risk appears more commonly among older cohorts^70,74^.

It remains possible that parity does exert durable effects prior to menopause, but these effects are too benign to be detected by clinical based measures of biological aging. Notably, our findings in pre-menopausal are in contrast to studies using measures of cellular aging, such as leukocyte telomere length and epigenetic age, which report evidence for a relationship between parity and accelerated cellular aging even among relatively young women^14,36,37^. Indeed, Pollack et al.^13^ found evidence for accelerated aging in response to parity in the form of shortened leukocyte telomere length in women 20–44 from the same dataset used here. Thus, cellular measures may provide early indicators of health impacts of parity that may only be detectable in post-reproductive years using clinical measures. Additional longitudinal studies investigating how these cellular measures of biological aging predict composite measures of biological aging as individuals age are therefore warranted.

The reproductive-cell cycle theory of aging offers a second potential explanation for parity-biological age acceleration relationships being present in post-, but not pre-menopausal, women. According to this theory, the protective forces acting to ensure survival during the reproductive stage of the lifespan are diminished in the post-menopausal period^44^. Changes in hypothalamic-pituitary–gonadal (HPG) axis functioning associated with menopause are proposed as the proximate cause of the increased physiological dysregulation observed in women in their post-reproductive stage. It is hypothesized that the combination of higher levels of hypothalamic and pituitary hormones, coupled with decreases in ovarian hormone production, together contribute to cell-cycle changes that then manifest as morbidity and mortality. Epidemiological and experimental lines of evidence support this hypothesis. Women who experience later menopause are at lower risk of cardiovascular disease, osteoporosis, and cognitive decline^75^, and menopausal status independent of age predicts biological age acceleration^76^, as was also found in the present study. Premenopausal women who undergo an oophorectomy (surgical removal of one or both ovaries) are at higher risk of these same outcomes^77,78^, suggesting the role of HPG axis outputs in modulating these age-related phenotypes. Experimental work manipulating ovarian hormone levels in animal models and observations of women taking hormone replacement therapy also find less age-related decline in hormonal milieus more closely approximating that of the reproductive stage (reviewed in^44^). However, it remains unclear precisely how changes in ovarian hormones associated with menopause contribute to cellular instability and aging. Thus, future work should explore different possible compensatory mechanisms buffering pre-menopausal from putative accelerated biological aging induced by parity and reproductive investment.

## Limitations

The fact that NHANES is cross-sectional rather than longitudinal in design contributes to two limitations in our study. First, its cross-sectional nature does not allow us to draw conclusions about causal relationships (or lack thereof). It is possible that accelerated biological aging increases reproductive effort in women, or a third unmeasured variable increases both biological aging and reproductive effort. This does not appear to be the case here, however, since there was no relationship between parity and biological age in pre-menopausal women. In the absence of longitudinal sampling, we also cannot be certain that biomarkers measured in this cross-sectional sample are not also representative of transient states unrelated to parity or reproduction. For example, it is possible that some participants could have been experiencing mild infections during MEC examinations, leading to altered clinical measures of immune function. Though this could contribute to imprecision in our biological aging measures, such imprecision would not be systematic and thus we would not expect it to significantly affect the present study’s findings.

Another limitation is our reliance on the relatively crude measures of reproductive effort in women. We were restricted to a measure of life births, but do not have access to data on miscarriages or aborted pregnancies, which could also be associated with costs of reproduction. We also lack information on breastfeeding, which is energetically costly in women^79^. Nevertheless, the fact that we do dectect a strong and robust signal of accelerated biological aging with parity in post-menopausal women implies that parity is adequate to capture important health-related costs in this population. Given the importance of hormones like estrogen in both reproduction and women’s health, it may also be important to include current use of oral contraceptives or hormone replacement therapy. Although NHANES collects data on lifetime patterns of hormonal contraceptive and hormone replacement therapy, it does not collect data on *current* use. Future studies assessing potential impacts of parity on biological age acceleration should thus consider effects of current hormone-altering medication use.

Finally, because data were collected in the United States, it is unknown whether similar patterns would be observed outside the context of WEIRD (Western, Educated, Industrialized, Rich, and Democratic)^80^ samples. WEIRD and non-WEIRD countries are characterized by significantly different activity patterns, nutrition, infectious disease ecology, and morbidity and mortality^81^, all of which could shape costs of reproduction. Whereas some studies have indeed examined links between parity and aging in non-Western settings^14,82^, more research is necessary to better catalogue and understand cross-cultural variation in costs of reproduction in women.

## Conclusions

We analyzed links between parity and different clinical-based measures of biological aging using a large, nationally-representative epidemiological sample of pre- and post-menopausal women in the United States. Our results are consistent with research in both historical populations and large epidemiological studies suggesting a nonlinear relationship between parity and health outcomes. Furthermore, our findings suggest these effects are only evident after menopause when indexed using these composite measures. This contrasts with measures with cellular aging, which appear to capture costs of reproduction in pre-menopausal women, suggesting the protective forces that work to prevent clinical level dysregulation induced by parity during the reproductive stage may be insufficient to dampen molecular level changes. Longitudinal studies are critically needed to evaluate molecular-based and clinical-based indices of biological age acceleration in tandem to better understand how costs of reproduction in women may manifest over time.

## Supplementary information

Supplementary Information.

## Data Availability

All script and data files that accompany this paper can be found at https://osf.io/b2jft/ (https://doi.org/10.17605/OSF.IO/B2JFT).
