# Supplementary material for: Parity predicts biological age acceleration in post-menopausal, but not pre-menopausal, women
Source: Sci Rep. 2020 Nov 25;10:20522. doi: 10.1038/s41598-020-77082-2 (PMC7689483; doi:10.1038/s41598-020-77082-2)
Supplement: Supplementary file 1 — Supplementary Information. [file 41598_2020_77082_MOESM1_ESM.docx]

**Parity predicts biological age acceleration but only in postmenopausal women**

Talia N. Shirazi^a*^, Waylon J. Hastings^b^, Asher Y. Rosinger^a,b^, Calen P. Ryan^c^

^a^ Department of Anthropology, Pennsylvania State University, University Park, PA, USA

^b^ Department of Biobehavioral Health, Pennsylvania State University, University Park, PA, USA

^c^ Department of Anthropology, Northwestern University, Evanston, IL, USA

Running title: Parity and biological age

* Corresponding author

Department of Anthropology

421 Carpenter Building

University Park, PA 16802

talia.shirazi@gmail.com

**ESM Text I. Regression equations for primary and sensitivity analyses**

**Primary model**

Predicted biological aging measure = b_0_ + b_1_(live births) + b_2_(live births^2^) + b_3_(age) + b_4_(BMI) + b_5_(BMI^2^) + b_6_(FIPR) +b_7_(smoking) +b_8_(education) + b_9_(ethnicity) + error

**Sensitivity analysis 1**

Predicted biological aging measure = b_0_ + b_1_(live births) + b_2_(live births^2^) + b_3_(age) + error

**Sensitivity analysis 2**

Predicted biological aging measure = b_0_ + b_1_(live births) + b_2_(live births^2^) + b_3_(BMI) + b_4_(BMI^2^) + b_5_(FIPR) +b_6_(smoking) +b_7_(education) + b_8_(ethnicity) + 9(years since last live birth) + b_10_(years since last live birth)(live births) + b_11_(years since last live birth)(live births^2^) + error

**Sensitivity analysis 3**

Predicted biological aging measure = b_0_ + b_1_(live births) + b_2_(live births^2^) + b_3_(months since last live birth) + b_4_(age) + b_5_(BMI) + b_6_(BMI^2^) + b_7_(FIPR) +b_8_(smoking) +b_9_(education) + b_10_(ethnicity) + b_11_(live births)(months since last live birth) + b_12_(live births^2^)(months since last live birth) + error

**ESM Table I.** Comparative demographics and pregnancy variables for sample of nonpregnant women aged 18-84 with complete biomarker data and all nonpregnant women aged 18-84.

| **ITEM** | **Women Aged 18-84 w/complete biomarker data (n = 5870)** | **All Nonpregnant Women Aged 18-84 (n = 13929)** | **Test Statistic** | **p-value** |
| --- | --- | --- | --- | --- |
| Mean Age (SD) | 48.52 (19.67) | 48.71 (20.09) | t_11244.681_ = -0.621 | 0.535 |
| Mean Live Births (SD) | 2.36 (2.07) | 2.34 (2.11) | t_17480_= 0.624 | p=0.532 |
| Proportion Non-Hispanic White | 47.34% | 47.32% | 𝟀_3_ = 3.339 | 0.342 |
| Proportion Hispanic | 28.71% | 27.81% |  |  |
| Proportion Non-Hispanic Black | 20.32% | 20.86% |  |  |
| Proportion Other Race Ethnicity | 3.63% | 4.01% |  |  |
| Proportion Less than HS | 29.70% | 30.30% | 𝟀_3_ = 1.644 | 0.649 |
| Proportion HS | 23.63% | 24.05% |  |  |
| Proportion Some College | 28.88% | 28.17% |  |  |
| Proportion College Graduate | 17.79% | 17.48% |  |  |
| Mean Poverty Income Ratio (SD) | 2.46 (1.60) | 2.41 (1.60) | t_18009_= 1.782 | p=0.075 |
| Percent Pre-menopause | 52.61% | 52.74% | 𝟀_1_ = 0.030 | 0.863 |
| Percent Post-menopause | 47.39% | 47.26% |  |  |
| Proportion Never Smoked | 59.59% | 60.02% | 𝟀_2_ = 0.786 | 0.675 |
| Proportion Past Smoker | 20.47% | 19.90% |  |  |
| Proportion Current Smoker | 19.95% | 20.08% |  |  |
| Proportion Never Pregnant | 17.92% | 19.32% | 𝟀_1_ = 4.793 | 0.029 |

**ESM Table II.** Ranges of biomarker values used to restrict the reference population for Homeostatic Dysregulation (HD) algorithm. *Note*: values from https://www.mayocliniclabs.com/test-catalog/Clinical+and+Interpretive/

|  | **Units of Measure** | **Range** |
| --- | --- | --- |
| Albumin | g/L | 35 - 50 |
| Creatinine | mg/dL | 0.59 - 1.04 |
| Glucose | mg/dL | <126 |
| CRP | mg/L | <3 |
| Lymphocyte Percent | % | 28 - 55 |
| Mean (red) cell volume | fL | 78.2 - 97.9 |
| Red Cell Distribution Width | % | 12.2 - 16.1 |
| Alkaline Phosphatase | U/L | 35 - 104 |
| White Cell Count | 1000 cell/uL | 3.4 - 9.6 |

**ESM Table III.** Descriptive statistics for the reference population (n = 481 women) used to define the Homeostatic Dysregulation (HD) algorithm.

|  | UNITS | **𝜇** | **𝜎** |
| --- | --- | --- | --- |
| Age | Yrs | 24.98 | 3.09 |
| Albumin | g/L | 41.57 | 3.23 |
| Creatinine | umol/L | 65.93 | 6.69 |
| Glucose | mmol/L | 4.81 | 0.38 |
| CRP | ln(mg/dL) | -1.36 | 0.47 |
| Lymphocyte Percent | % | 38.19 | 6.35 |
| Mean cell volume | fL | 88.55 | 3.98 |
| Red cell width | % | 12.98 | 0.68 |
| Alkaline Phosphatase | U/L | 66.93 | 15.38 |
| White Cell Count | 1000 cell/uL | 6.28 | 1.42 |

**ESM Table IV.** Biomarker variance covariance matrix for the reference population used to define the Homeostatic Dysregulation (HD) algorithm. *Note:* Biomarkers were standardized to mean=0, standard deviation=1 prior to computing the variance covariance matrix.

|  | Albumin | Creatinine | Glucose | Ln(CRP) | Lymphocyte Percent | Mean Cell Volume | Red Cell Width | Alkaline Phosphatase | White Cell Count |
| --- | --- | --- | --- | --- | --- | --- | --- | --- | --- |
| Albumin | 1.00 | 0.05 | -0.07 | -0.22 | -0.04 | 0.20 | 0.00 | -0.01 | 0.12 |
| Creatinine | 0.05 | 1.00 | -0.06 | -0.03 | 0.07 | 0.02 | -0.03 | -0.01 | -0.04 |
| Glucose | -0.07 | -0.06 | 1.00 | 0.02 | -0.07 | 0.00 | -0.03 | 0.06 | -0.07 |
| Ln(CRP) | -0.22 | -0.03 | 0.02 | 1.00 | -0.05 | -0.05 | 0.02 | 0.10 | 0.09 |
| Lymphocyte Percent | -0.04 | 0.07 | -0.07 | -0.05 | 1.00 | 0.00 | 0.09 | -0.15 | -0.28 |
| Mean Cell Volume | 0.20 | 0.02 | 0.00 | -0.05 | 0.00 | 1.00 | -0.38 | -0.05 | 0.02 |
| Red Cell Width | 0.00 | -0.03 | -0.03 | 0.02 | 0.09 | -0.38 | 1.00 | 0.08 | -0.05 |
| Alkaline Phosphatase | -0.01 | -0.01 | 0.06 | 0.10 | -0.15 | -0.05 | 0.08 | 1.00 | 0.16 |
| White Cell Count | 0.12 | -0.04 | -0.07 | 0.09 | -0.28 | 0.02 | -0.05 | 0.16 | 1.00 |

**ESM Table V.** Biomarker descriptive statistics for the reference population used to build the Klemera-Doubal Method (KDM) biological age algorithm (n = 5,453). *Note:* The reference sample consisted of non-pregnant women aged 30-75 participating in NHANES III.

|  | **Units** | **Mean** | **SD** |
| --- | --- | --- | --- |
| Age | Yrs | 49.76 | 13.59 |
| Albumin | g/L | 40.46 | 3.37 |
| Creatinine | umol/L | 65.92 | 14.20 |
| Glucose | mmol/L | 5.76 | 2.16 |
| Ln(CRP) | ln(mg/dL) | -1.02 | 0.76 |
| Lymphocyte Percent | % | 33.94 | 8.43 |
| Mean cell volume | fL | 88.79 | 5.65 |
| Red cell width | % | 13.25 | 1.13 |
| Alkaline Phosphatase | U/L | 86.38 | 28.69 |
| White Cell Count | 1000 cell/uL | 7.15 | 2.10 |

.

**ESM Table VI.** KDM biological age model parameters estimated from NHANES III reference sample. We report the intercept (Q), slope (K), and root mean squared error (S) for the regressions of biomarkers onto chronological age in the NHANES III reference sample. R_char_ refers to a ‘characteristic correlation’ describing a summary relationship between the biomarker panel and chronological age. S_BA_^2^ corresponds to the squared variance in chronological age explained by the biomarker panel in the reference sample.

|  | **Intercept** | **Slope** | **RMSE** |
| --- | --- | --- | --- |
| Albumin | 41.545 | -0.022 | 3.361 |
| Creatinine | 52.070 | 0.279 | 13.836 |
| Glucose | 4.094 | 0.033 | 2.114 |
| Ln(CRP) | -1.228 | 0.004 | 0.766 |
| Lymphocyte Percent | 34.673 | -0.015 | 8.426 |
| Mean cell volume | 86.302 | 0.050 | 5.615 |
| Red cell width | 13.077 | 0.004 | 1.135 |
| Alkaline Phosphatase | 55.182 | 0.628 | 27.385 |
| White Cell Count | 7.528 | -0.007 | 2.101 |
| r_char_ | 0.277 | | |
| S_BA_^2^ | 870.150 | | |

**ESM Table VII**: Risk Thresholds for Allostatic Load Construction

| **Biomarker (unit)** | **At Risk Threshold** |
| --- | --- |
| Albumin (g/L) | ≤ 40.00 |
| Creatinine (umol/L) | ≥ 75.70 |
| Glucose (mmol/L) | ≥ 5.93 |
| Ln(CRP) (ln(mg/dL) | ≥ -0.511 |
| Lymphocyte Percent | ≥ 36.1 |
| Mean Cell Volume (fL) | ≥ 92.7 |
| Red Cell Width (%) | ≥ 13.30 |
| Alkaline Phosphatase (U/L) | ≥ 83.00 |
| White Cell Count (1000 cell/uL) | ≥ 7.90 |

**ESM Table VIII.** Biomarker and biological age summary statistics for the final analytical sample (n = 4,418).

|  | **Units** | **Mean** | **SD** |
| --- | --- | --- | --- |
| Age | Yrs | 50.31 | 18.41 |
| Albumin | g/L | 41.45 | 3.05 |
| Creatinine | umol/L | 69.10 | 16.37 |
| Glucose | mmol/L | 5.81 | 1.51 |
| Ln(CRP) | ln(mg/dL) | -1.34 | 1.20 |
| Lymphocyte Percent | % | 30.76 | 8.28 |
| Mean Cell Volume | fL | 89.18 | 5.66 |
| Red Cell Width | % | 12.90 | 1.16 |
| Alkaline Phosphatase | U/L | 70.73 | 23.80 |
| White Cell Count | 1000 cell/uL | 6.75 | 1.89 |
| LM Biological Age | Yrs | 46.43 | 19.79 |
| KDM Biological Age | Yrs | 46.32 | 20.56 |
| Ln(HD) |  | 3.20 | 0.50 |
| Allostatic Load |  | 0.27 | 0.17 |

**ESM Table IX.** Correlations between biological aging measures after adjustment for chronological age. Note: * *p* < 0.05,  ** *p* < 0.01, *** *p* < 0.001

|  | LM (adjusted) | HD log (adjusted) | KDM  (adjusted) | AL (adjusted) |
| --- | --- | --- | --- | --- |
| Age | 0.00 | 0.00 | 0.00 | 0.00 |
| LM (adjusted) |  | 0.34*** | 0.66*** | 0.64*** |
| HD log (adjusted) |  |  | 0.48*** | 0.36*** |
| KDM (adjusted) |  |  |  | 0.60*** |
